# Transperitoneal Laparoscopic Pyelolithotomy in Pelvic Ectopic Kidneys: Experience From a Northern Indian Tertiary Care Institution

**DOI:** 10.7759/cureus.65406

**Published:** 2024-07-26

**Authors:** Manish K Aggarwal, Sashiraj Singh, Vishwajeet Singh, Mukul K Singh

**Affiliations:** 1 Urology, King George's Medical University, Lucknow, IND

**Keywords:** pyelolithotomy, stone, pelvic, ectopic, laparoscopic, transperitoneal

## Abstract

Introduction

The ectopic pelvic kidneys have a higher likelihood of developing renal stones due to urinary stasis caused by the abnormal position of the renal pelvis, altered course of the ureter, and kidney malrotation. This retrospective study highlights the safety, efficacy, and feasibility of performing transperitoneal laparoscopic pyelolithotomy in cases of pelvic ectopic kidney.

Methodology

The 15 patients with ectopic pelvic kidneys and nephrolithiasis underwent laparoscopic pyelolithotomy. The kidney was exposed either by moving the bowel or using a trans-mesocolic approach. A surgical procedure was performed to remove stones from the renal pelvis using laparoscopic forceps. Following the placement of a double J stent, the incision in the renal pelvis was closed. The procedure was completed after the intraperitoneal drain was inserted.

Results

A total of 15 patients underwent the transperitoneal laparoscopic pyelolithotomy procedure, with a male-to-female ratio of 3:2. The average age of the patients was 41 (25-58) years, while the average size of the stones was 3.8 cm. Additionally, seven (46.6%) patients had the presence of caliceal stones in conjunction with the pelvic stone. Out of the 15 patients, some had stones on the left side (*n* = 9, 60%), while others had stones on the right side (*n *= 6, 40%). The operation with an average duration was 125 minutes with a range of (90-190). Fourteen (93.3%) patients were found to be free of stones. A patient required extracorporeal shock wave lithotripsy (ESWL) to address a small caliceal residual stone measuring 8 mm. After just one session of ESWL, this stone was completely cleared. All stones were successfully removed, resulting in a 100% stone-free rate.

Conclusions

Laparoscopic pyelolithotomy is a highly effective and efficient procedure for treating large and numerous stones in the ectopic pelvic kidney. This method has a significant level of efficiency in removing stones with limited consequences.

## Introduction

The abnormally located kidney in the pelvis is a rare congenital anomaly with an incidence rate of 1/2,200 and 1/3,000 [1]. These pelvic kidneys that are located outside of their normal position are at a higher risk of developing renal stones. This is due to the urine not flowing properly because of the position of the renal pelvis, the altered course of the ureter, and the kidney being rotated incorrectly [2]. Treatment options for pelvic kidney stones include open surgery, extracorporeal shock wave lithotripsy (ESWL), percutaneous nephrolithotomy (PCNL), retrograde intrarenal surgery (RIRS), and laparoscopic surgery. Still, the best modality of treatment for ectopic pelvic kidney stones is under debate [3].

The open pyelolithotomy has limitations as open surgery has high morbidity due to long incisions and scar marks, the need for more bowel mobilization, increased postoperative pain, and hospital stay. The ESWL is an effective option for stone disease but not suitable for ectopic pelvic kidneys particularly with large stone burden. As there is abnormal drainage of the pelvic calyceal system in such cases, there is a reduced stone-free rate and increased risk of leaving residual fragments [4]. RIRS is also not suited due to difficulty in negotiating a flexible ureteroscope through a tortuous ureter in an ectopic kidney.

Eshghi et al. first described the laparoscopy-assisted PCNL for the treatment of pelvic kidney stones [5]. Laparoscopic pyelolithotomy has gained popularity in recent years due to its very high stone-free rate, lower risk of bleeding, and fewer chances of injury to nephrons in comparison to PCNL [6]. Soltani et al. conducted laparoscopic pyelolithotomy in a patient with staghorn stones in an ectopic pelvic kidney and concluded that the procedure was a safe and effective treatment modality and also proposed it as a first-line management option [7].

However, limited literature is available on laparoscopy pyelolithotomy in ectopic pelvic kidney stones [7-10]. Most of the studies are in the form of a case study or small case series. Our paper reviews the experience, regarding the safety and efficacy of laparoscopy pyelolithotomy in ectopic pelvic kidneys with renal calculi.

## Materials and methods

This study was conducted in the Department of Urology at King George’s Medical University in Lucknow from January 2012 to December 2023. It was a retrospective observational study. Our study received ethical approval from the Institutional Ethics Committee (Registration No. ECR/262/Ins/2012/RR-16) and adheres to the declaration of Helsinki. An analysis was conducted on the medical records of patients who had pelvic kidneys with stones. Information about the disease and its treatment was recorded from the hospital database. Transperitoneal laparoscopic pyelolithotomy was performed on 15 patients with ectopic pelvic kidneys and renal calculi. Patients who had pelvic kidneys located outside their normal position and had one or more stones larger than 1 cm were included in the study.

Patients who had ectopic pelvic kidney with renal calculi smaller than 1 cm, bleeding diathesis, were unfit for surgery, or did not give consent for the procedure were excluded based on specific criteria.

**Figure 1:**
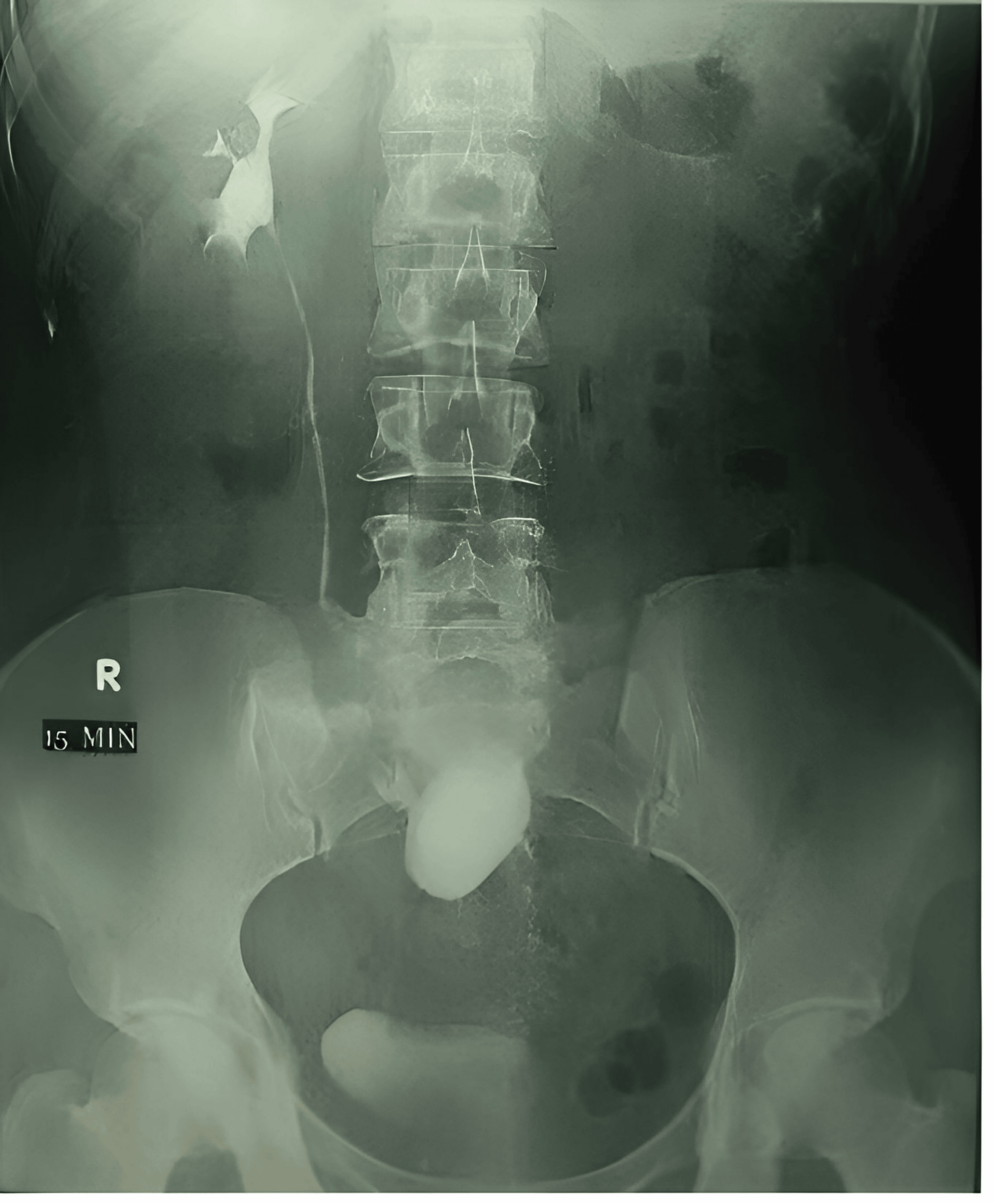
Laparoscopic pyelolithotomy for recurrent stones in a previously operated ectopic pelvic kidney.

The records were obtained retrospectively and consisted of medical history, physical examination, standard hematological and blood chemistry tests, urine investigations, ultrasonography of the kidney, ureter, and bladder (USG KUB), and intravenous urogram (IVU) or computed tomography (CT) urography (Figure 1). The study analyzed multiple variables of the patients, including their age, gender, location of the pelvic kidney, size and position of the stone, length of the surgical procedure, duration of the double J (DJ) stent in place, duration of the abdominal drain, and length of hospital stay. Complications noticed and reported during the intraoperative or postoperative phase were duly considered.

Details of the operative procedure

After induction of general anesthesia, the patient was positioned in lithotomy and cystoscope guided a ureteric catheter was placed under fluoroscopic guidance. Then the patient was placed in the Trendelenburg position with ipsilateral side, 30 degrees up for the laparoscopic approach. A Veress needle insertion was done to create a pneumoperitoneum. A 10-mm laparoscopic trocar port was placed at the level of the umbilicus. Two 5-mm trocars were inserted, one in the midline, midway between the umbilicus and the symphysis pubis, and another one at the lateral border of the rectus muscle. In some patients, a third 5 mm trocar was placed for retraction, at the anterior axillary line and placed laterally and distally to the last port. The small bowel loops were retracted and kept away from the pelvis. The ectopic kidney was localized in the pelvis. For the right-side ectopic kidney, the right colon was mobilized and dissected off from the anterior surface of the kidney. A trans-mesocolic approach was preferred to expose the anterior surface of the left-sided kidneys having dilated pelvis with thin mesocolon/mesentery or similar to the right side the left side colon was reflected to expose and locate the kidney.

Injection of saline via the ureteric catheter was used to identify and localize the renal pelvis. The wall of the renal pelvis was exposed after dissecting perirenal fat over the pelvis. Then an incision was given to the renal pelvis using both hot and cold scissors. The stones were removed using laparoscopic forceps and placed in the peritoneal cavity which was removed at the end of the procedure. After removal of all the stones, a DJ stent (5/20 French) was placed followed by the closure of renal pelvic incision by 3-0 polyglactin using running or interrupted intracorporeal sutures. In all cases following stone removal and DJ placement, an on-table X-ray using fluoroscopy was performed to see the position of the stent and if any residual fragments lying in the pelvicaliceal system. The stones were then removed from the peritoneal cavity in an indigenously made endo-bag via a 10-mm port site, which was enlarged a little bit by giving an incision at the margin. A 20-French soft silicone transperitoneal drain was placed under camera guidance for postoperative drainage, and the procedure was completed. All patients received broad-spectrum antibiotic coverage postoperatively for three to five days. DJ stent removal was done after two to four weeks. All patients were followed up with plain radiography (X-ray KUB) and ultrasonography KUB at six months.

## Results

**Figure 2:**
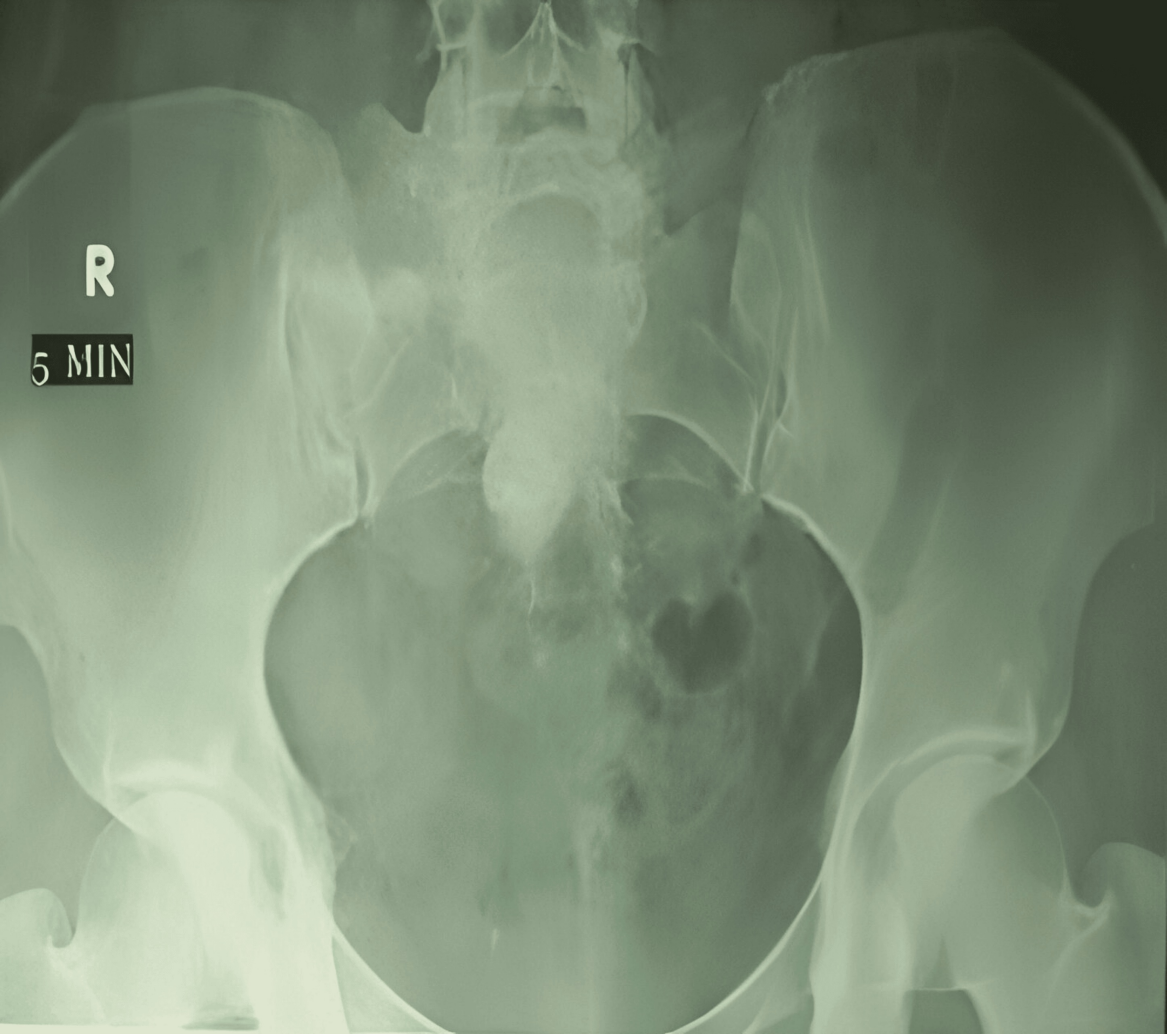
Intravenous urogram (IVU) of preoperative patients.

**Figure 3:**
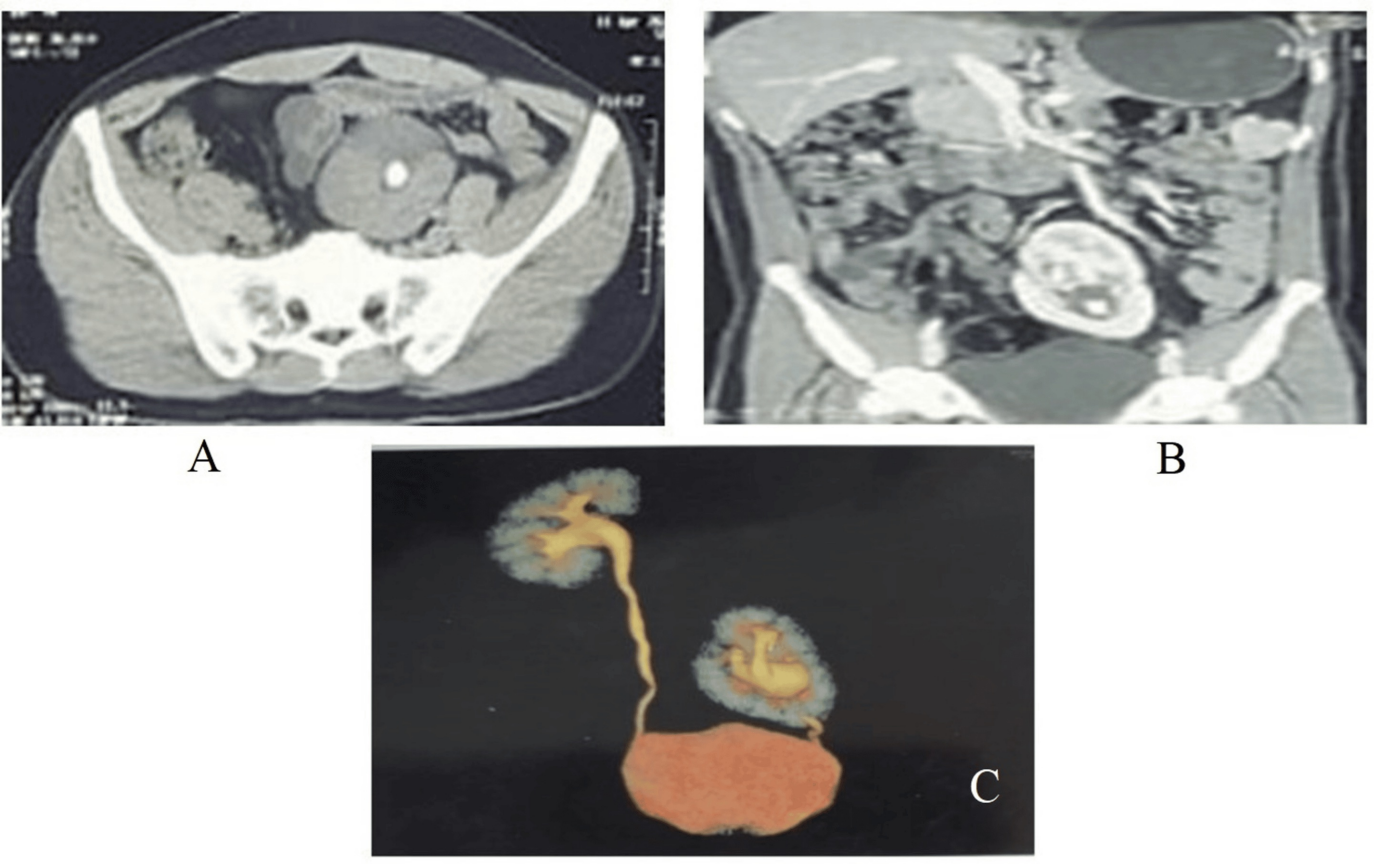
(A) Preoperative CECT KUB in a patient with a single large stone in an ectopic left-sided kidney and (B) shows urography. CECT KUB, contrast-enhanced computed tomography of the kidneys, ureters, and bladder

A total of 15 patients with pelvic kidneys (male:female ratio 9:6), with a mean age of 41 years (range 25-58 years) underwent successful transperitoneal laparoscopic pyelolithotomy. All procedures were completed without conversion to open surgery or intraoperative complications. Nine pelvic kidney stones were on the left and six on the right side (right:left ratio 9:6), as shown in Table 1 and Figures 1-3.

**Table 1:** Stone size, stone location, fluoroscopy time, hospitalization time, and demographic characteristics of patients.

Patients characteristics		Number (*N *= 15)
Age (years)		41 (25–58)
Sex	Male	9
	Female	6
Stone size (cm), Mean (Range)		3.8 (2.2-5.4)
Stone side	Left	9
	Right	6
Stone location	Renal pelvis	15
	Upper calyces	3
	Middle calyces	2
	Lower calyces	2
Mean (Range), operative time (minutes)		125 (90-190)
Stone-free rate (%)		100
Mean fluoroscopy time (seconds)		18 (10-30)
Mean hospitalization time (days)		4.5 (4-7)

**Figure 4:**
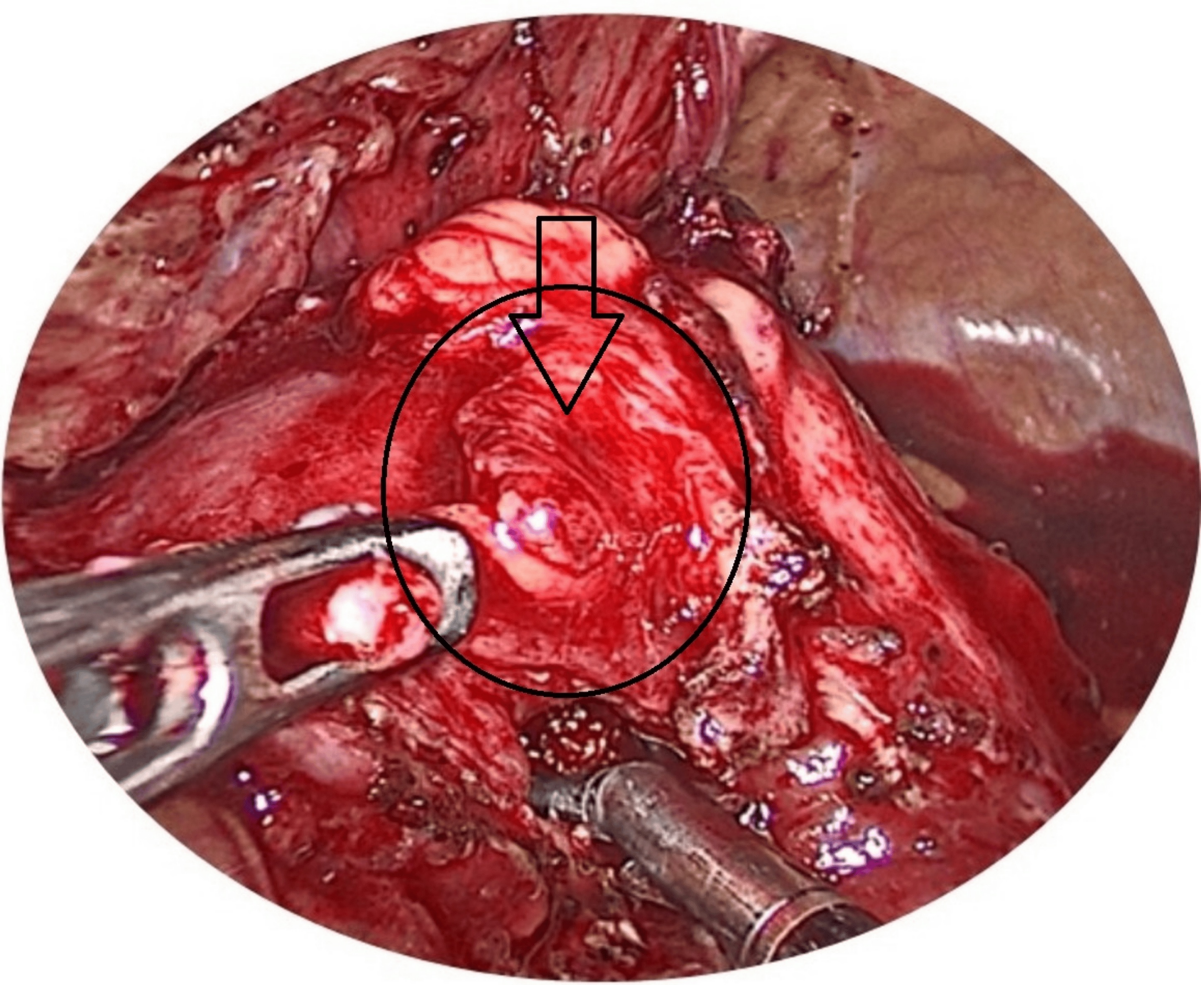
Laparoscopic image showing the opened renal pelvis with a stone.

**Figure 5:**
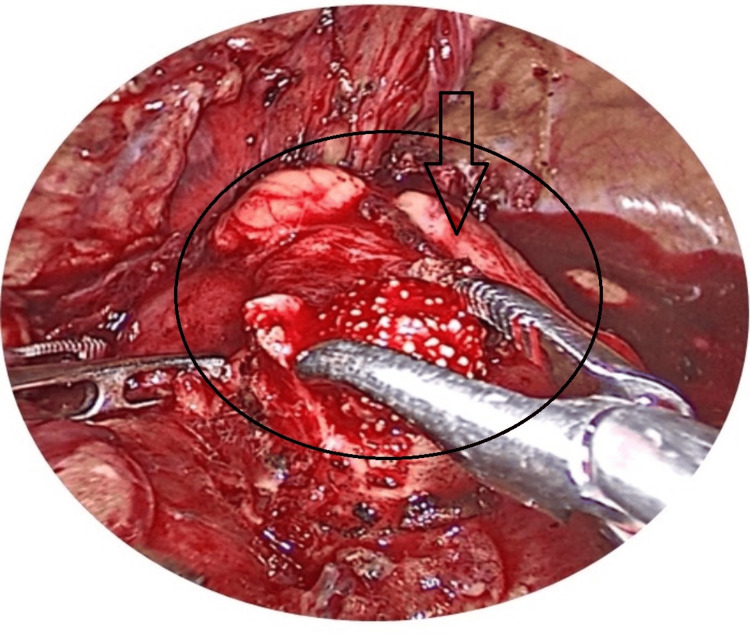
Laparoscopic image showing the stone being held with forceps.

**Figure 6:**
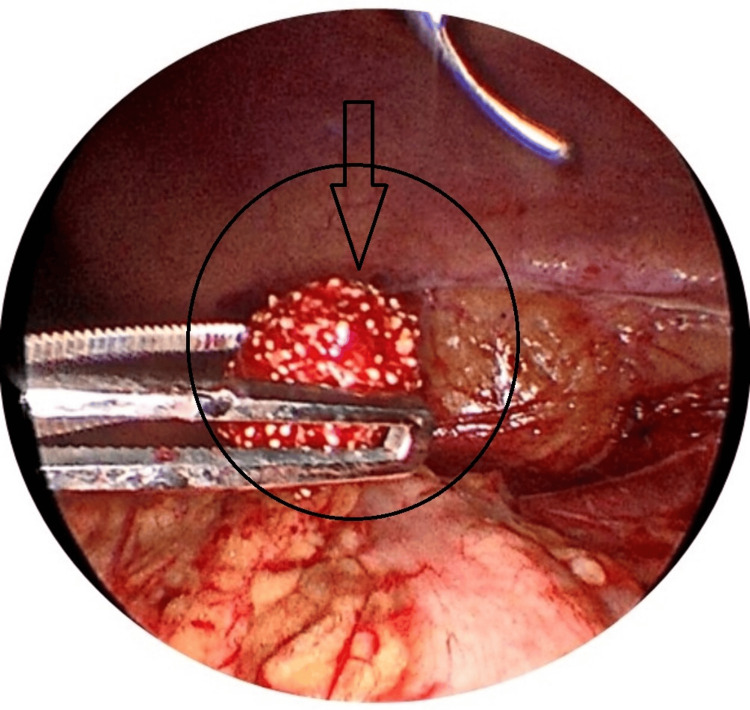
Laparoscopic image showing stone retrieval.

A trans-mesocolic approach was used to expose the posterior peritoneum covering the anterior wall of the kidneys in six of the nine patients with left-sided disease. In the remaining three patients, the kidney was accessed by reflecting the left-sided colon. On the right side, in all six patients, the kidneys and renal pelvises were exposed by reflecting the right colon. None of the patients experienced intraoperative vascular or bowel injury. One patient had a postoperative temporary ileus that lasted 48 hours, which was managed conservatively (Figures 4-6).

In this series, the number and location of stones and their distribution were as follows: single (renal pelvic) in seven (46.7%) patients, two stones (pelvic and caliceal) in 3 (20%) patients, and multiple stones (pelvic and/or caliceal) in four (26.6%) patients. None of the patients had any concomitant ureteric stones. The average stone size was 3.8 cm (2.2-5.4 cm). The mean operative time was 125 minutes (90-190 minutes).

The renal pelvis was closed by 3-0 polygalactin as interrupted sutures in all patients. On postoperative day 2, plain X-ray KUB and USG KUB were performed. Fourteen (93.3%) patients were stone-free following laparoscopic pyelolithotomy. In one patient, an 8-mm residual caliceal stone was missed intraoperatively. This caliceal stone was managed two weeks following surgery by a single session of ESWL. The mean hospital stay was 4.5 days (range 4-7 days).

The DJ stent was left for an average of three weeks (2-4 weeks). In a mean follow-up of 12 months (3-40 months), none of the patients showed a recurrence of stone and or significant dilatation of the pelvicaliceal system.

## Discussion

The ectopic pelvic kidney is a rare entity with an increased risk of renal obstruction and nephrolithiasis. Patients with such conditions may need combined endourologic procedures to make kidney stone-free. ESWL, though the least invasive method for treating stones in ectopic pelvic kidneys, is associated with a lower stone-free rate compared to ESWL in normal kidneys. ESWL in the pelvic kidney has a success rate of only about 54% [11]. RIRS is also an option, but it is associated with technical difficulties due to ureteral kinking [12]. The most commonly employed method for stone clearance in ectopic pelvic kidneys is percutaneous renal surgery. Overall PCNL, either alone or with laparoscopic-assisted, is an acceptable option because of the stone-free rate. In pelvic kidneys, there are technical difficulties mainly related to access. There is also a potential risk of nerve damage [13]. Standard PCNL requires a larger tract, which may be unsafe in a kidney with an anomalous blood supply [14].

Laparoscopic pyelolithotomy for the management of stones in ectopic pelvic kidneys is acquiring significance. The transperitoneal approach is feasible in such kidneys as it directly visualizes abnormal anatomy, abnormal vasculature, and related structures [15,16]. The kidney is approached by mobilizing the ipsilateral colon or via a trans-mesocolic approach [13]. For right-sided cases, mobilization of the colon is easy, and it allows good exposure to the kidney. In the present study, none of the patients among right-sided cases required additional trocar for bowel retraction. For left-sided cases, our preferred approach to expose the kidney was the trans-mesocolic approach, which allowed access through the thin peritoneum over the distended pelvis with stones. In three patients with left-sided ectopic kidneys, our approach involved reflecting and medially displacing the colon to expose the kidney. In these cases, there was excessive fat deposition over the renal pelvis, and the pelvicaliceal system was not distended.

Gupta et al. in a series of six patients with ectopic pelvic kidneys revealed that the laparoscopic method is safe and suitable for nephrectomy, pyeloplasty, and pyelolithotomy [17]. In the present study, 15 patients with stones in ectopic pelvic kidneys were treated successfully using the laparoscopic approach. In our study, the stone-free rate was 93.3% but it reached 100% after one session of ESWL in one patient who had a small residual fragment that missed following the laparoscopic procedure. In our selected cohort of patients, all had either single or multiple stones in the pelvis with proximally dilated calyces, which permitted caliceal stone removal. The mean operative time is 125 minutes, and the dissection of abnormally located kidneys takes time to finish the procedure. The stents were removed under local anesthesia and Intravenous sedation at OPD visits.

Limitations

This study has some limitations, including its retrospective nature and small sample size.

## Conclusions

Laparoscopic pyelolithotomy by transperitoneal route is a suitable and plausible treatment modality for single or multiple calculi in an ectopic pelvic kidney. It has a high stone-free rate without any significant postoperative complications.
